# Overlapping Neural Systems Represent Cognitive Effort and Reward Anticipation

**DOI:** 10.1371/journal.pone.0091008

**Published:** 2014-03-07

**Authors:** Eliana Vassena, Massimo Silvetti, Carsten N. Boehler, Eric Achten, Wim Fias, Tom Verguts

**Affiliations:** 1 Department of Experimental Psychology, Ghent University, Ghent, Belgium; 2 Ghent Institute for Functional and Metabolic Imaging, Ghent University Hospital, Ghent, Belgium; University Medical Center Groningen UMCG, Netherlands

## Abstract

Anticipating a potential benefit and how difficult it will be to obtain it are valuable skills in a constantly changing environment. In the human brain, the anticipation of reward is encoded by the Anterior Cingulate Cortex (ACC) and Striatum. Naturally, potential rewards have an incentive quality, resulting in a motivational effect improving performance. Recently it has been proposed that an upcoming task requiring effort induces a similar anticipation mechanism as reward, relying on the same cortico-limbic network. However, this overlapping anticipatory activity for reward and effort has only been investigated in a perceptual task. Whether this generalizes to high-level cognitive tasks remains to be investigated. To this end, an fMRI experiment was designed to investigate anticipation of reward and effort in cognitive tasks. A mental arithmetic task was implemented, manipulating effort (difficulty), reward, and delay in reward delivery to control for temporal confounds. The goal was to test for the motivational effect induced by the expectation of bigger reward and higher effort. The results showed that the activation elicited by an upcoming difficult task overlapped with higher reward prospect in the ACC and in the striatum, thus highlighting a pivotal role of this circuit in sustaining motivated behavior.

## Introduction

Reward processing has been investigated by several disciplines, ranging from economics to psychology and machine learning [1]. An established finding is that animals typically strive for the most beneficial consequences of their action, and that they do so via optimizing the net reward they can obtain from the environment [2]. This complex skill relies on reward estimation, which is precisely encoded in the primate and in the human brain [3]–[7]. This consists in anticipating the value of the potential benefit. Nevertheless, benefits seldom come for free. They usually entail some cost, and this cost is taken into account by the brain to calculate the net value of each available option [8]–[11]. Usually, obtaining a benefit requires a certain degree of effort, either in terms of cognitive demand [12] or physical energy expenditure [13]–[15]. The more effortful the task, the less the animal values the respective reward [16], [17]. Humans also discount reward by effort [18], [19], meaning that subjective reward value decreases as a function of the effort required to obtain it. Hence, also effort needs to be estimated when calculating reward value, and a major role in this process has again been attributed to the Anterior Cingulate Cortex (ACC) and the striatum. These structures would integrate predicted cost and reward in a net value signal [8], [9].

Besides estimating reward and cost, expecting to earn a reward is a powerful motivational factor per se [20]. This can improve behavioral performance [21] and influence learning and memory, according to a concept known as incentive-salience [20], [22]. At the neural level, the anticipation of a potential reward is associated with increased activation in the ACC and striatum [5].

Recent evidence suggests that facing an upcoming effortful task also induces increased ACC and striatum involvement. This might reflect a motivational effect towards task performance, comparable to the incentive given by a monetary reward [23]–[25]. In terms of energy expenditure, this would be translated to the invigoration of the optimal behavior, which in turn is required to obtain a reward. Several findings in animals support this hypothesis, identifying its neural mediator in the fronto-striatal dopaminergic system [26]. Accordingly, if this circuit is pharmacologically inhibited [27] or lesioned [13] the ability of engaging in a high-demand task to obtain a reward is blunted. A recent fMRI study in humans [28] also highlighted the contribution of this network in anticipating higher energy expenditure, in terms of a more effortful grip.

Thus, both reward and effort anticipation are core functions ascribed to ACC and striatum [4], [5], [28]. How and whether these elements are combined when cognitive effort is required, recently received considerable attention [18], [19], [29]–[31]. However, findings concerning ACC and striatum are controversial. Krebs et al. [23] made a first attempt towards clarifying this matter, by combining reward and effort in an attentional-cueing paradigm in order to probe for shared neural activation. In that study, both task demand (effort) and reward were manipulated in a perceptual task. The cue predicting the more effortful condition elicited a stronger activation of the midbrain and striatum, dopaminergic structures that broadly innervate the ACC [32]. Moreover, this nigro-striatal network partially overlapped with the activations elicited by the cue predictive of a high reward, and the ACC maximally responded to the high reward/high effort condition. These results are interpreted by the authors as part of a resource-recruitment process, essential in successfully accomplishing the task and hence obtaining the reward. Nevertheless, this result was obtained in a perceptual task where during the preparation period the allocation of attentional resources was crucial for successful completion. It is unclear if this finding extends to tasks requiring higher-level cognitive skills, thus relying on a more general preparation effect. This would argue in favor of a motivational effect, going beyond attentional-cueing facilitation. The contribution of the ACC in preparation for arithmetical tasks [33] and in logical-rules tasks [34] would strongly suggest this mechanism to be a more general preparation effect, in line with theories of task-set preparation [35], [36], rather than a simple spatial-attention facilitation. However, this hypothesis has never been tested in demanding high-level cognitive tasks in combination with reward.

Hence, an fMRI experiment was designed where cognitive effort and reward prospect were manipulated in order to investigate effort and reward anticipation. The goal was to test for the cognitive equivalent of a behavioral invigoration signal, especially in the ACC and in the striatum.

Moreover, a third condition was added, where the delay in reward delivery was manipulated. Controlling the time variable is crucial, as effortful tasks typically require more time to be performed. Delay estimation is in fact a well-known mechanism both at the behavioral and the neural level [37]–[40] which in the light of the current purpose could be a potential confound. For these reasons the same task was implemented for both effort and delay conditions. Furthermore, this allowed to test the specificity of the motivational effect of the effort condition.

In the experiment, in each trial the cue phase informed about the upcoming reward, effort, or delay. The task consisted of solving arithmetic operations of different degrees of difficulty. In a first step, the anticipatory encoding of high-level cognitive effort and reward was tested, as well as their overlap [23]. This aimed at determining the type of encoding of these two variables. A motivational encoding would imply higher activation for higher effort and bigger reward, as those would serve as incentive to task performance. An alternative encoding would be value-related, where maximal response should be reported for the condition with the highest net-value (low effort and big reward). This putative shared substrate was also tested.

In a second step, selective response to the anticipation of cognitive effort was addressed in an exploratory analysis, to isolate a potential neural mechanism specifically supporting cognitive effort exertion, unrelated to reward.

## Materials and Methods

### Participants

Twenty-five healthy volunteers participated in this experiment (8 males). Three subjects were excluded from further analyses due to excessive head motion (more than 3 mm motion in either rotation or translation). This left 22 subjects (8 males), with a mean age of 20 (range 18–24). The experimental protocol was approved by the Ethical Committee of the Ghent University Hospital. All participants signed an informed-consent form before the experiment, and confirmed they had no neurological or psychiatric history.

### Experimental Procedure

**Figure 1:**
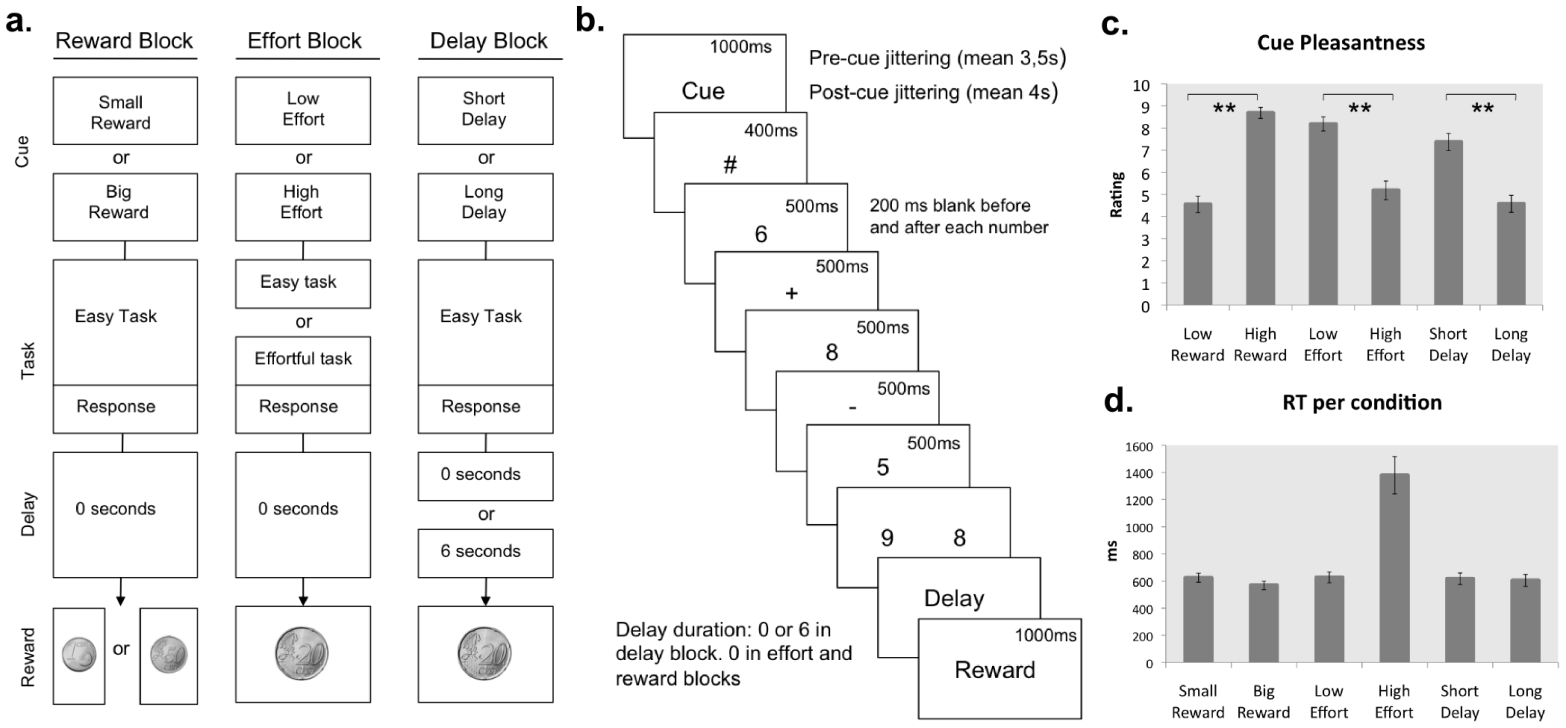
Task structure and behavioral performance. a. Block types. In every block only one trial type is presented, where only one feature is manipulated. In a trial in the reward block, the cue informs about the final reward being small or big. In a trial in the effort block, the cue informs about the difficulty level (low or high). In the delay block, the cue informs about the length of the delay between response and reward delivery (short or long). b. Task structure and timing. The cue presentation is followed by a fixation symbol. The task follows, consisting of an addition followed by a subtraction. Two possible results are presented and the subject has to choose the correct one. After the response, a delay can occur. If the response was accurate, the reward is shown. c. Average rating of pleasantness for every cue-type (small reward cue, big reward cue, low effort cue, high effort cue, short delay cue, long delay cue). e. Average reaction times (RTs) in every condition (small reward, big reward, low effort, high effort, short delay, long delay). RT in the high effort condition is significantly higher than in the low effort condition (p<0.001).

An event-related fMRI design was set up, with the main manipulations being separated into different experimental blocks. In every block, reward, effort, or delay was manipulated, resulting in three different block types (Figure 1a). Every block type was presented twice, resulting in six randomized blocks in the experiment. To avoid sequence effects, a block type was never preceded or followed by the same block type. Every block started with a display informing the participant about the block type (reward, effort, or delay block).

Every trial in a block started with a cue formed by two words, informing participants whether the manipulated feature (reward, effort, or delay) would be low or high (Figure 1a). The resulting six cues were “Small Reward”, “Big Reward”, “Low Effort”, “High Effort”, “Short Delay”, or “Long Delay”. Within a block, the presentation of different trial types (i.e. “Low Effort” and “High Effort”) was randomized. The inter trial interval (ITI) was randomly jittered (range 2–5 seconds, mean 3.5) as well as the period between cue onset and task onset (range 2–6 seconds, mean 4, Figure 1b). At task onset, two subsequent arithmetic operations had to be performed, an addition followed by a subtraction. Participants had to mentally perform the calculation and then select the correct solution from two possible results by pressing the corresponding key (Figure 1b). Correct responses were followed by positive feedback consisting of a picture of a coin representing the reward. Errors were followed by the word “incorrect”.

In the reward condition, the reward could be small or big, leading to a win of 1 cent or 50 cents after performing the easy version of the task, with no delay in reward delivery. In the effort condition, the task could be easy or difficult. In both cases it consisted in single digit calculations, but in the difficult condition every single operation required carrying or borrowing, whereas the easy condition did not [41]. In this case the reward was constant at 20 cents, and there was no delay in delivery. In the delay condition, the interval between response selection and reward delivery could be short (no delay) or long (6 seconds). The task was easy and the reward constant at 20 cents. The cues were fully predictive of the manipulation, thus ruling out uncertainty confounds. Trials in the reward and effort blocks lasted on average 14 seconds, while trials in the delay block lasted on average 17 seconds. The experiment consisted of 180 trials in total (60 trials per condition, 30 trials per event), with each condition divided in two blocks. The participants underwent a short version of the experiment as training before the scanning session. They were asked to be as fast and as accurate as possible. At the end of the experiment, they received the amount of money that they won by performing the calculations.

We focused our analyses on the cue period activity, thus avoiding potential confounds of actual effort, motor response activation, or differential delay. The experiment was implemented in E-prime 2.0 (www.pstnet.com/eprime; Psychology Software Tool) and presented to the participants using a dual display MRI compatible LCD display and mounted in a lightweight headset (VisuaStim XGA, Resonance Technology Inc., Northridge, CA; http://www.mrivideo.com/).

### Ratings and Questionnaires

Participants filled in a safety checklist prior to scanning and a post-scan checklist after the session. Every block was followed by a short break, in which the participant was asked to rate how much attention he had paid to the cues. These questions aimed at keeping the participant focused on the cue and avoiding potential distractions. At the end of the session participants filled in two more questionnaires. One questionnaire queried the pleasantness of each cue type and the pleasantness of the effective outcome related to each cue, in order to check whether the high cost options were perceived as less pleasant. The second questionnaire was the Bis/Bas [42], testing reward sensitivity, drive and fun-seeking tendencies.

### fMRI Data Acquisition

Images were acquired through a 3 T Magnetom Trio MRI scanner (Siemens), using an 8 channel radio frequency head coil. First, an anatomical T_1_ weighted sequence was applied, collecting 176 high-resolution slices (TR = 1550 ms, slice thickness = 0.9 mm, voxel size = 0.9′0.9′0.9, FoV = 220 mm, flip angle = 9°). Subsequently, functional images were acquired using a T_2_* weighted EPI sequence (30 slices per volume, TR = 2000 ms, slice thickness = 3 mm, distance factor = 17%, voxel size = 3.5×3.5×3.0, FoV = 224 mm, flip angle = 80°). The session was divided into 6 runs. On average 225 volumes per run were collected. Run length varied according to the block type, namely 7 minutes for reward blocks and effort blocks and 8.5 minutes for delay blocks.

### fMRI Data Analysis

After discarding the first 4 volumes of each run to allow for steady-state magnetization, data were preprocessed with SPM8 (http://www.fil.ion.ucl.ac.uk/spm). Images were realigned to the first image of each run and the structural image was coregistered to the functional mean image to allow a more precise spatial normalization. The unified segmentation and nonlinear warping approach of SPM8 was applied to normalize structural and functional images to the MNI template (Montreal Neurological Institute). Functional images were then smoothed with a Gaussian kernel of 8 mm full width half maximum (FWHM).

Subsequently a General Linear Model (GLM) was applied in order to identify each subject’s condition-specific activations. Cue onsets were modeled as events of interest (2 regressors per run) and two condition-specific task regressors (from stimulus onset to response, 2 regressors per run) were introduced to account for task- and motor-related activation. Four further regressors were added to model trials in which errors were made (2 cue-locked regressors plus 2 task-locked regressors) in order to exclude them from the contrasts of interest. The resulting stimulus functions were convolved with the canonical hemodynamic response function. To account for low frequency noise a 128 s high pass filter was included; to account for serial auto-correlation, an autoregressive model was applied. All group-level effects are based on random-effects analysis.

First, contrasts of interest were computed at the group level, generating a Reward contrast (big reward>small reward), an Effort contrast (high effort>low effort) and a Delay contrast (long delay>short delay). The reversed contrasts for effort and delay were also computed, in order to test for preferential activation for low cost anticipation (low effort>high effort, short delay>long delay). The voxel-level threshold was set to 0.001 uncorrected. A whole-brain cluster-level family-wise error (FWE) correction for multiple comparison was applied, with a p-value of 0.05.

Second, we performed a conjunction between single contrasts (strict conjunction approach [43] ((big reward>small reward) & (high effort>low effort)). The goal of this contrast was to test for shared neural activation in reward and effort anticipation. A whole-brain cluster-level FWE correction for multiple comparison with a p-value of 0.05 was applied to each component.

Third, in order to isolate the neural response selective to high effort, the following contrast was performed: (high effort – low effort)>(big reward – small reward)). This would reveal effort-related activity, when controlling for response to reward. On the basis of previous findings, reporting a significant contribution of the brainstem nuclei in different types of effortful conditions [23]–[25], [44]–[46] and in response to high-arousal situations [47], a small volume correction (SVC) for the brainstem region was applied to this contrast, to test for brainstem involvement. Within this volume, we applied a voxel-level threshold of 0.001 uncorrected, with a cluster-level FWE correction for multiple comparison (p-value 0.05). It should be noted that this was an exploratory analysis, as the current protocol would not grant sufficient spatial resolution to separate different brainstem nuclei.

## Results

### Behavioral Performance

As predicted, a repeated-measures ANOVA on the reaction times (RTs) revealed a significant interaction between condition (reward, effort, delay) and cue-type (low, high; F_(2, 42)_ = 47.2, p<.001).

Pairwise comparisons across participants revealed a significant difference in the high effort compared to the low effort condition (t_(21)_ = 6.874, p<0.001, Figure 1d). In particular, subjects were significantly faster in performing easy than difficult calculations (difference of 760 ms). This confirms the effectiveness of the effort manipulation. As expected, for the delay and reward condition, no significant difference was found between the two cues (long vs. short delay, p = 0.88; big vs. small reward p = 0.33).

Overall accuracy was very high (average 98%). In the effort block, average accuracy was also calculated for low effort (98%) vs. high effort trials (96%). This small difference was however significant (t_(21)_ = 2.13, p = .045), confirming that the high effort trial were more difficult to perform than the low effort trials. Despite being very small, this difference might carry the potential confound of uncertainty estimation, as the chance of successful completion of a high-effort trial was slightly smaller for some participants. Although it seems unlikely that this difference in accuracy might have confounded the anticipation of effort, the dissociation between effort anticipation and uncertainty estimation should definitely be investigated in future research.

### Ratings

Pairwise comparisons on the ratings about the pleasantness of the cues were performed to ensure that effort and delay costs were actually perceived as unpleasant. Indeed at the end of the experiment the participants rated the big reward cue as significantly more pleasant than the small reward cue (t_(21)_ = 9.14, p<.001), the low effort cue as more pleasant than the high effort cue (t_(21)_ = 6.87, p<.001) and the short delay cue as more pleasant than the long delay cue (t_(21)_ = 5.53, p<.001, see Figure 1c).

Furthermore, the pleasantness ratings for the big reward cue correlated with the reward responsiveness scale of the Bis/Bas (r = .49, p<.01), indicating that more reward-responsive participants also liked the big reward cue more.

Participants were asked to provide ratings during every break, quantifying how much attention they had paid to the cues during the previous block, on a scale from 1 to 10. The goal of these ratings was to keep participants focused on the cues. A one-way repeated-measures ANOVA on the scores with cue type as a factor (reward, effort, delay) revealed a significant difference (F(2,42) = 19.7, p<.001). Pair-wise comparisons showed that participants paid more attention to the reward cues (M = 6.73, SD = 2.08) as compared to the delay cues (M = 4.59, SD = 2.53, t_(21) = _4.36, p<.001) and to the effort cues (M = 7.59, SD = 1.83) as compared to the delay cues (t_(21) = _6.05, p<.001). The difference between reward and effort cues was not significant ((t_(21) = _−1.76, p = .09). These ratings suggest that while reward and effort cues were correctly attended to, overall participants paid less attention to the delay cues.

### fMRI Results

**Figure 2:**
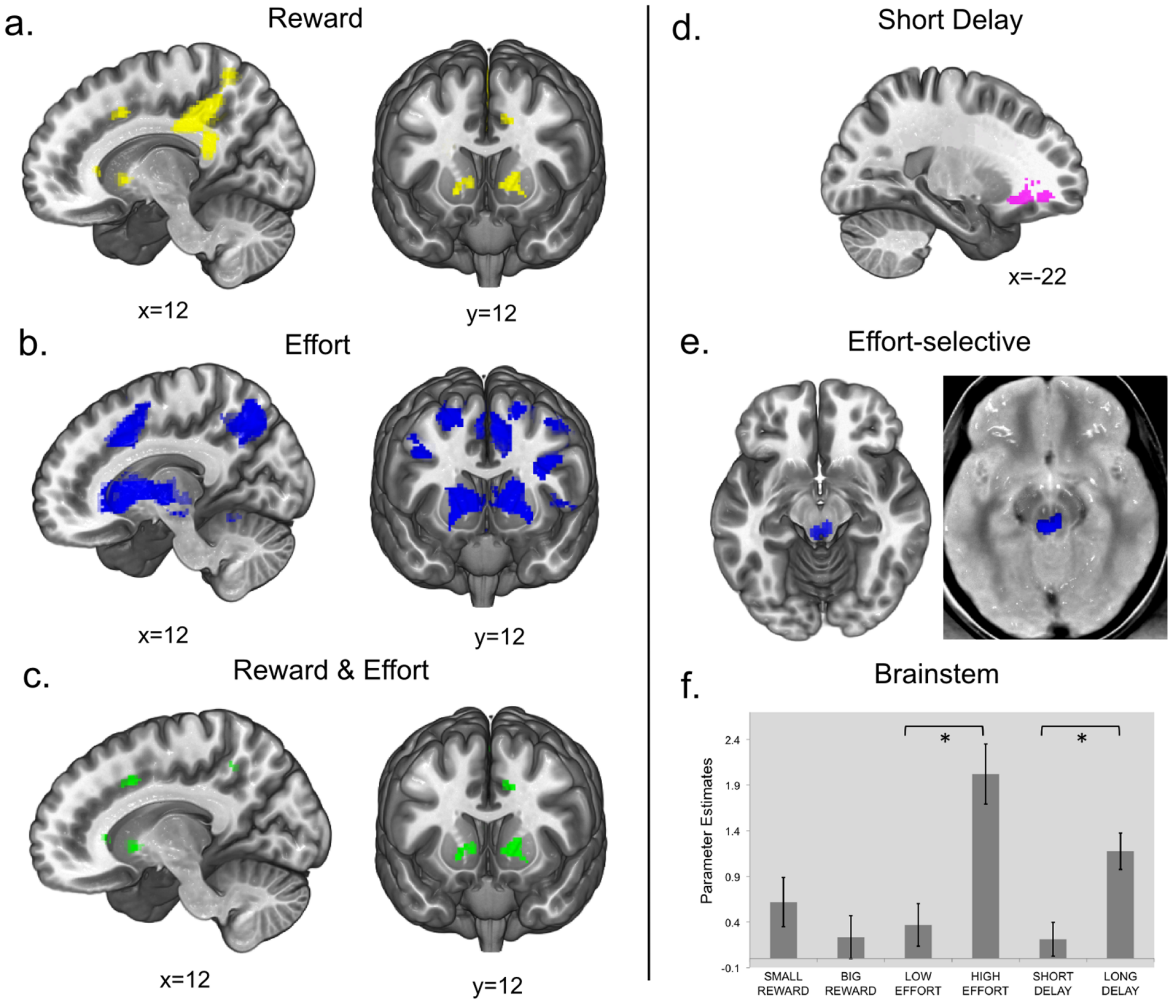
fMRI Results. a. Reward contrast (big reward>small reward). Activation clusters are located in the posterior cingulate cortex (PCC), anterior cingulate cortex (ACC), striatum and superior frontal gyrus (SFG). b. Effort contrast (high effort>low effort). Activation clusters are located in the posterior cingulate cortex (PCC), brainstem, anterior cingulate cortex (ACC), insula, striatum and middle frontal gyrus (MFG). c. Conjunction of high effort>low effort & big reward>small reward. Overlapping activation clusters are located in the striatum, anterior cingulate cortex (ACC) and precuneus. d. Short delay>long delay contrast. The activation cluster is located in the left orbitofrontal cortex (OFC). e. Effort-selective activation ((high effort>low effort)>(high reward> low reward)), SVC for the region of the brainstem, p value 0.05 FWE correction for multiple comparisons, plotted on Proton Density Weighted MRI Template (left image). f. Parameter estimates plot at voxel −4, −32, −10 (MNI coordinates), local maximum in the activation cluster located in the Brainstem in the effort-selective contrast.

First, the single contrasts during the cue period were computed (see Table 1 for a summary). The Reward contrast (big reward>small reward, Figure 2a) showed significant activation in the left caudate nucleus, right anterior cingulate (ACC) and right posterior cingulate cortex (PCC). Then, anticipation of effort was addressed (high effort>low effort, Figure 2b). This contrast resulted in widespread activation, originating a cluster of 27430 voxels. Such an extended cluster-size might hamper the validity of the cluster-level inference [48], especially concerning regional specificity. For this reason a more stringent voxel-level threshold was applied (uncorrected p = 0.0001 instead of the standard 0.001). This resulted in breaking down the massive cluster in multiple clusters, thus ensuring a better localization of the significant activations. Anticipation of effort significantly activated striatum bilaterally, left brainstem, right ACC, supplementary motor area (SMA), primary mortor cortex bilaterally, left premotor cortex, left Insula, right superior frontal gyrus (SFG) and precuneus bilaterally. The Delay contrast (long delay>short delay) did not show any significant activation cluster surviving the whole brain FWE threshold correction.

**Table 1:** Summary of the activation clusters in the whole-brain contrasts.

	Local Maxima	Cluster	Peak	cluster-level
Area	MNI Coordinates	size	T	p(FWE-cor)
*Big Reward >Small Reward*				
Posterior Cingulate Cortex	18 −40 34	3574	5.54	0.000
Thalamus	0 −18 18		4.31	
Inferior Parietal Cortex	−38 −28 30	598	4.33	0.001
Left Striatum	−10 14 2	290	3.78	0.026
Precuneus	6 −52 62		4.54	
Superior Frontal Gyrus	24 42 16	786	4.19	0.000
Right Striatum	22 28 2		4.12	
Anterior Cingulate Cortex	20 20 34		4.04	
High Effort>Low Effort (*)				
Left Striatum	−8 6 2	6574	6.43	0.000
Brainstem	−2 −28 −20		5.94	
Right Striatum	10 10 −2		5.86	
Right primary motor cortex	40 −2 40		5.77	
Anterior Cingulate Cortex	8 12 46		5.35	
Superior Frontal Gyrus	20 8 62		5.29	
Right Precuneus	18 −68 38	1631	5.81	0.000
Inferior Parietal lobule	32 −50 46		5.09	
Left Precuneus	−8 −72 38	543	5.57	0.000
Premotor cortex	−24 6 60	478	5.28	0.000
Left primary motor cortex	−38 6 36	358	5.19	0.000
*Short Delay>Long Delay*				
Orbitofrontal Cortex	−22 44 −8	243	4.75	0.047
*Effort-selective contrast (SVC)*				
Brainstem	−4 −32 −10	129	4.00	0.010

(*) voxel-level threshold p = 0.0001 uncorrected.

Legend: p(FWE)-cor = cluster-level family-wise error corrected p-values. SVC = small volume corrected. For regions including multiple local maxima, the highest local maximum is reported.

In the reversed Effort contrast (low effort>high effort) no clusters survived the whole brain threshold. Concerning the reversed Delay contrast (short delay>long delay) the orbitofrontal cortex (OFC) proved to be sensitive to shorter delay (Figure 2d).

Second, the strict conjunction between effort- and reward-related activation ((high effort>low effort) & (big reward>small reward); incentive conjunction) revealed activation in the striatum bilaterally, the precuneus bilaterally and the right ACC (Figure 2c, see Table 2 for a detailed list).

**Table 2:** List of regions resulting from the overlap of the Reward and Effort contrast, thus responding to both anticipation of high effort and big reward.

Conjunction		
High Effort>Low Effort & Big Reward>Small Reward		
	Local Maxima	Cluster
Area	MNI Coordinates	size
	x y z	
Left Precuneus	−8 −72 38	260
Right Striatum	10 10 −2	171
Right Precuneus	8 −54 48	133
Left Striatum	−14 10 −4	97
Anterior Cingulate Cortex	12 14 40	49

As a third step, the effort-selective contrast ((high effort >low effort) – (big reward>small reward)) showed a selective involvement of the brainstem in effort anticipation (Figure 2e, T_(21)_ = 4,00, p = 0.01, SVC). No clusters at the cortical level survived. For exploratory purposes, the brainstem activated cluster was superimposed on a high-resolution proton-density averaged template normalized to the MNI space, as this sequence allows identifying the Substantia Nigra (SN) [49] thereby providing a reference for better anatomical characterization of the brainstem (Figure 2e). At visual inspection, the location of the activation cluster is not consistent with the main dopaminergic nuclei. According to the Duvernoy’s atlas [50], the location of this cluster might be compatible with other non-dopaminergic brainstem nuclei, including the serotonergic Dorsal Raphe Nucleus (DRN), or the noradrenergic Locus Coeruleus (LC). The parameter estimates for every condition for the peak voxel of this cluster are plotted in figure 2f. Paired comparisons performed on these scores revealed a significantly higher response for high effort as opposed to low Effort (T_(21)_ = −3.73, p = .001) and for long delay as opposed to short delay (T_(21)_ = 2.891, p = .009). No differential response was detected for high reward as opposed to low reward (T_(21)_ = −1.033, p = .313). Given its potential theoretical relevance, this exploratory result is further discussed below, yet one should note the exploratory nature of this result. It should also be noted that the resolution of the current fMRI protocol was not optimal to distinguish between different small structures in the brainstem.

## Discussion

The present study investigated the anticipation of high-level cognitive effort required to obtain a reward, while controlling for temporal confounds. Crucially, both prospective effort and reward anticipation activated the same network, involving the ACC and the striatum. This confirms the contribution of these areas to incentive-motivation and supports the essential role of this network in sustaining task-preparation for cognitive effort. The current results do not find support for a value-related encoding, according to which low effort should have elicited a stronger response. Moreover, exploratory analyses suggest a selective contribution of the brainstem to cognitive effort anticipation.

Reward-related activation (Figure 2a) was identified in the ACC and striatum, principal targets of dopaminergic midbrain projections [32] and key components of reward circuitry [4], [10], [51], [52]. Also, the right PCC was activated in this condition, which is known to be selectively activated by monetary gain anticipation compared to primary reinforcers [53].

The anticipation of a higher cognitive effort (Figure 2b) activated the bilateral striatum, right ACC and left brainstem, among other regions. Preparing to perform difficult calculations seems to rely on the same system that subserves other demanding cognitive functions, such as conflict monitoring [34], [54] working memory encoding [55], and top-down attentional facilitation [23], [24]. This converging evidence confirms the role of the ACC not only in experiencing effort [56], but also for effort anticipation during task preparation [18], [29]–[31]. The information of an upcoming demanding task seems to act as a motivational factor needed for successful task completion. This would be in line with theoretical accounts of task preparation and task-set maintenance [35], [36], [57]. This preparation effect might be mediated via dopaminergic transmission, which would be consistent with the hypothesized role of dopamine in invigorating behavior [23], [58] in effortful tasks. In the context of a task where effort is required to obtain a reward, dopaminergic release may enhance motivation for performing effortful actions, in order to overcome response cost and reap the expected benefit [16]. A potential mechanism is that motivational stimuli, such as the prospect of reward, boost the neuronal signal-to-noise ratio towards optimal performance [59]. A similar underlying mechanism might be called upon in the case of a prospective difficult task.

This interpretation finds support in animal experiments, where dopaminergic depletion induces effort avoidance [60], [61]. A convergent computational framework has also been suggested by Niv et al. [58], where dopaminergic neurotransmission would be crucial in mediating response vigor.

Dopaminergic mediation of behavioral invigoration has also been confirmed in a pharmacological study in humans [62]. fMRI experiments in humans demonstrated the involvement of the ACC and the striatum in the anticipation of physical effort [28] or perceptual load [23]. The current results show that this mechanism supports high-level cognitive effort as well, in line with what was proposed by Sohn et al. [34].

Accordingly, ACC activity has been proven to be influenced by fatigue deriving from sustained effort in cognitive tasks [63], [64]. Moeller et al. showed that prolonged performance under taxing cognitive requirements is associated with decreased ACC activation and as a consequence, reduced error-related responses. This supports a key role of this region in successfully enacting cognitive effortful behavior. Interestingly, the authors also showed how this pattern is altered in cocaine-abusers, known to have abnormal dopamine levels, and how this effect can be reversed by administering a dopaminergic-agonist medication. These results together converge on the underlying dopaminergic mediation of cognitive demanding task requirements.

Interestingly, cognitive effort anticipation recruits a cortico-subcortical network that partially overlaps with reward-related regions, as shown in the conjunction analysis (Figure 2c). This confirms the hypothesized motivational effect which might reflect higher engagement induced by both the prospect of a greater benefit and the expectation of a difficult task. In this perspective, both high effort and high reward cues induce a stronger preparation effect, translated into increased neural recruitment of areas coding for incentive. For the first time, this result is shown in a high-level cognitive task, suggesting that ACC and striatum contribute to an incentive-induced resource allocation. Further converging indications are supplied by a recent study with Positron Emission Tomography (PET), that showed a correlation between dopamine release in the striatum and subjective willingness to exert effort in exchange of a reward [65]. The fronto-striatal network seems therefore to be crucial in supporting reward-driven effort exertion. The putative dopaminergic nature of this mediation is also in line with previous evidence showing the crucial influence of dopamine on high-level cognitive processes [66]. Moreover, these findings are compatible with a recently proposed view of ACC function [67]. Here, the authors formalize the contribution of this region as estimator not only of the amount of control to be exerted (effort in our case), but also of the value of exerting control, in so far as it leads to a rewarding outcome.

In the same contrast, the precuneus was also activated bilaterally. The contribution of this region to the anticipation of both effort and reward offers interesting ground for further investigation.

Subsequently, an exploratory analysis was performed investigating selective response to cognitive effort anticipation but not to reward prospect. Given previous evidence reporting a contribution of the brainstem and theories suggesting a role for brainstem neuromodulatory systems [23], [24], [44]–[47], an SVC was applied for the volume of the brainstem to test for its involvement. The contrast testing selective response to effort ((high effort>low effort)>(big reward>small reward)) isolated an effort-selective signal in the brainstem (Figure 2e). Definitive anatomical inference on this region cannot be performed on the current data, given the resolution constraints. It is however possible to speculate on the nature of this activation. The cluster location is not consistent with locations usually reported for midbrain dopaminergic nuclei in fMRI studies [23], [24], [68]. The current location might be compatible with other brainstem structures, like the serotonergic Dorsal Raphe Nucleus (DRN) or the noradrenergic Locus Coeruleus (LC; Figure 3a and 3b). These hypotheses might deserve further investigation, given that previous evidence suggests a potential contribution of these nuclei in aversive processing and arousal. On the one hand, a wealth of studies demonstrated striking effects of manipulating serotonin levels on processing aversive events [69]–[74]. In this perspective, expecting an upcoming effort might be considered aversive (as confirmed in our task by the ratings) and therefore rely on serotonergic midbrain input to blunt aversiveness or related behavioral reactions, and perhaps boost prefrontal activity needed for accurate task perfomance [35], [75], [76]. On a convergent note, theoretical and computational frameworks of cost and benefit encoding have assigned a putative function to serotonergic modulation [77], [78]. On the other hand, anticipating higher effort might induce an arousal response and therefore elicit noradrenaline release [47], [80], thus suggesting that the present functional result would reflect putative LC-noradrenergic activity. Convergent evidence for a putative LC contribution during demanding tasks was also provided by Raizada and Poldrack [44]. At the current stage, both hypotheses are rather speculative. This result might however be informative and fruitful ground for further investigation.

As for the additional experimental condition, the delay manipulation, the expectation of a short delay (short delay>long delay, Figure 2d) revealed a value-related signal in the orbitofrontal cortex, consistent with evidence from delay discounting studies [80], [81]. No significant activation was elicited by the prospect of a longer delay. The exploratory analysis on the brainstem activation however, shows a stronger response in that region not only for greater efforts, but also for longer delays (Figure 2e). With the caveat of the localization limitation, it is worth nothing that a critical involvement of the DRN in delay discounting has been recently shown in rats, where serotonergic activity seems to facilitate waiting for a benefit [82], and to be necessary to tolerate longer delays (7–11 seconds) [83]. Additional evidence is accumulating supporting the hypothesis of serotonin involvement in promoting a more foresighted reward evaluation in both animals and humans [81], [84]–[86]. Considering the methodological limitations of the current experiment, this might be fruitful venue for future research.

## Conclusion

This study provides the first evidence for a shared motivational effect induced at the neural level by both reward prospect and the anticipation of cognitive effort in complex cognitive tasks. This is associated with activation in the ACC and the striatum, supporting behavioral engagement and resource-recruiting towards a final goal. Moreover, an exploratory analysis identified an effort-selective signal in the human brainstem, which suggests potential contribution of non-dopaminergic brainstem nuclei to effort anticipation.
